# Emotional factors, medical interventions and mode of birth among low-risk primiparous women in Poland

**DOI:** 10.1093/emph/eoad013

**Published:** 2023-05-14

**Authors:** Ilona Nenko, Katarzyna Kopeć-Godlewska, Mary C Towner, Laura D Klein, Agnieszka Micek

**Affiliations:** Department of Environmental Health, Faculty of Health Sciences, Jagiellonian University Medical College, Krakow, Poland; Laboratory of Fundamental Obstetric Care, Faculty of Health Sciences, Jagiellonian University Medical College, Krakow, Poland; Department of Integrative Biology, Oklahoma State University, Stillwater, OK, USA; Business Growth and Innovation, Australian Red Cross Lifeblood, Alexandria, Australia; Department of Nursing Management and Epidemiological Nursing, Faculty of Health Sciences, Jagiellonian University Medical College, Krakow, Poland

## Abstract

**Background and objectives:**

Birth is a critical event in women’s lives. Since humans have evolved to give birth in the context of social support, not having it in modern settings might lead to more complications during birth. Our aim was to model how emotional factors and medical interventions related to birth outcomes in hospital settings in Poland, where c-section rates have doubled in the last decade.

**Methodology:**

We analysed data from 2363 low-risk primiparous women who went into labor with the intention of giving birth vaginally. We used a model comparison approach to examine the relationship between emotional and medical variables and birth outcome (vaginal or c-section), including sociodemographic control variables in all models.

**Results:**

A model with emotional factors better explained the data than a control model (Δ_AIC_ = 470.8); women with continuous personal support during labor had lower odds of a c-section compared to those attended by hospital staff only (OR = 0.12, 95% CI = 0.09 − 0.16). A model that included medical interventions also better explained the data than a control model (Δ_AIC_ = 133.6); women given epidurals, in particular, had increased odds of a c-section over those who were not (OR = 3.55, 95% CI = 2.95 − 4.27). The best model included variables for both the level of personal support and the use of epidural (Δ_AIC_ = 598.0).

**Conclusions and implications:**

Continuous personal support during childbirth may be an evolutionarily informed strategy for reducing complications, including one of the most common obstetrical complications in modern hospital settings, the c-section.

## 1. INTRODUCTION

Cross-culturally, women are accompanied by others during birth, historically by female family members [1], especially during their first birth [2]. Birth assistance likely evolved as an adaptation to difficult and complicated birth in humans [3]. Birth in modern humans is thought to be particularly complicated due to the anatomical configuration of our pelvis, which is adapted to bipedal locomotion, and the relatively large head of the baby [1], but also see [4]. Moreover, human infants usually rotate as they pass through the birth canal and are typically born facing away from the mother (an occiput anterior position), which makes it difficult, though not impossible, for the woman to give birth by herself [5, 6]. Although giving birth being surrounded by others was for years thought to be a human-specific trait, we now know that other primates also give birth being watched by others [5]. Other primates, however, will only rarely assist a female in labor by directly touching the mother or infant [5]. Instead, it seems that the presence of conspecifics may reduce the anxiety and stress of a parturient. For modern humans, the help of others to guide the baby from the birth canal may be especially important for a safe birth for both the mother and baby and could lead to ‘obligate midwifery’ [3, 6].

In addition to facilitating the expulsion of the baby from the birth canal, such midwives could also help support birthing women emotionally through what may be a long and painful process. Studies show that being alone during labor raises feelings of fear and anxiety [7] and that women benefit from and value having someone with them who can provide physical, emotional and informational support [8]. For example, women from Botswana who were accompanied by a female relative had more frequent spontaneous vaginal births, fewer medical interventions during birth and fewer vacuum extractions and cesarean sections (c-sections) than unaccompanied women [9]. By helping reduce the stress of hospitalization, a support person may reduce the mother’s cortisol levels, whereas high cortisol inhibits the release of endogenous oxytocin and may prolong labor [10, 11]. A recent study of primiparous women in Sweden found that continuous support by a midwife was associated with lower cortisol during the first and second stages of labor, which was correlated with shorter active labor [12]. Moreover, continuous support was followed by spontaneous delivery in 73%, instrumental delivery in 24% and emergency c-section in 3% of births; in contrast, intermittent support was followed by spontaneous delivery in 62%, instrumental delivery in 24% and c-section in 14% of births.

C-section rates are increasing worldwide [13]. A c-section can be life-saving when medically indicated [14], but it is also a major surgical operation that can have short- and long-term health consequences both for the mother [15–21] and baby [22–26]. After a c-section (both planned and emergency), there is a higher risk of severe acute maternal morbidity than after planned vaginal birth [14]. According to the World Health Organization (WHO), c-section rates above 15% at the population level are not associated with reductions in maternal and newborn mortality rates [27]. Among countries in the Organization for Economic Co-operation and Development (OECD) in 2017, c-section rates were lowest in Israel, Norway, Iceland, the Netherlands and Finland, ranging from 14.8% to 16.5% of all live births [28]. In contrast, rates were highest in Turkey, Korea, Chile, Mexico and Poland, ranging from 39.3% to 53.1% of all live births. Suggested reasons for increasing c-section rates in developed countries include sociodemographic factors such as women’s later ages at first birth and higher prevalence of obesity [29, 30], which may elevate health risks during pregnancy and birth, leading to more c-sections. While the medical need in developed countries for c-sections is influenced by changing demographic trends in who gives birth and when, these factors are unlikely to fully explain the recent increase in c-sections. For example, cesarean rates in Poland rose rapidly in the last decade from below 20% in 2007 to 39.3% in 2017 [28]. One major contributor to this trend is that vaginal births after c-sections are rare, such that having had a previous c-section elevates the risk in all subsequent births, and is, in fact, the most common reason reported for a current c-section in Poland [31]. We, therefore, need a better understanding of the determinants of c-sections among women with low-risk pregnancies who are giving birth, particularly for the first time.

From a public health perspective, it is critical to identify a wide range of risk factors for cesarean births in order to enable healthcare specialists to avoid unnecessary interventions. Recently, more attention has been paid to the role of emotional factors such as fear of childbirth [32] or satisfaction with pregnancy care in rising c-section rates [33]. Other factors such as style of professional practice, fear of pain and organizational, social and cultural factors have also been implicated [34]. Our aim in this study was to examine whether emotional factors such as level of support during labor and medical interventions (e.g. epidural) were associated with non-elective c-sections among low-risk primiparous women with singleton pregnancy in Poland. Although c-sections are a modern surgical outcome, they are also a proxy measure for obstetrical difficulty more generally, particularly among women with low-risk pregnancies intending to give birth vaginally. We hypothesize that women who have continuous personal support are likely to have lower rates of c-section births.

## 2. METHODOLOGY

### 2.1 Participants

Data on pregnancies and birth were collected from women via an online survey widely promoted on social media in November–December 2017. The questionnaire was disseminated to groups dedicated to women and parents, famous Polish parenting bloggers, as well as through the Polish Association of Lactation Consultants and popular web portals. A pilot study was conducted (*n* = 10) to ensure clarity of the questions. Although online, self-administered questionnaires have limitations, benefits include accessing a large, geographically dispersed sample that would be prohibitive for in-person surveys. Ethics approval of the study protocol was granted by the Bioethics Committee at Jagiellonian University and consent was obtained from all participants.

In the full dataset, we have collected pregnancy and birth experience data from 9836 women on 14,810 pregnancies (range 1–7, mean 1.8). For the present study, we examined responses from 2363 low-risk primiparous women (aged 18–40 years) with singleton pregnancies who were qualified for vaginal birth based on their health status before and during pregnancy. All women gave birth in public hospitals and went into labor with the intention of giving birth vaginally, but depending on how labor progressed, delivered either vaginally (*N* = 1650; 69.8%) or by c-section (*N* = 713; 30.2%).

Compared to national data for Poland, our study sample has a moderately lower c-section rate (30.2% vs. 39.3% in 2017), although the national data are not limited to first-time mothers [28]. In terms of representativeness of our study sample more generally, women are quite similar in age at first birth compared to national levels (median: 28 years vs. 27.8 years) [35]. Our sample includes women living in diverse locations (small cities and villages as well as larger cities) and with all levels of education, but does include more women with higher completed levels of education, as would be expected given the sampling method [35].

### 2.2 Variables

Table 1 summarizes our study variables. The dependent variable in all analyses was the mode of birth, either vaginal or c-section. We grouped our predictor variables into three categories: Controls, Medical Interventions and Emotional Factors. For Controls, we considered both demographic and social predictors, including the woman’s age at birth, childbirth year, pregnancy length, education level, marital status and place of residence. Controls were selected based both on known patterns (e.g. temporal changes in c-section prevalence) and standard demographic methods (e.g. controlling for age). Emotional Factors included whether the pregnancy was planned, fear of childbirth and support during labor. Medical Interventions included the use of epidural anesthesia (epidural), oxytocin and amniotomy. Apart from demographic controls, all variables were categorical (refer to Table 1 for levels).

**Table 1.** Descriptive statistics of studied women (*N* = 2363)

		Vaginal births (*n* = 1650, 69.8%)	C-sections (*n* = 713, 30.2%)	Full sample (*n* = 2363)
	***Continuous variables***	median (mean, SD, range)		
**Demographic Controls**	*Age at birth (years)*	28 (27.5, 3.6, 18–40)	28 (28.0, 3.6, 18–40)	28 (27.7, 3.6, 18–40)
	*Childbirth year*	2016 (2016, 1.9, 2000–2017)	2016 (2016, 2.0, 2000–2017)	2016 (2016, 1.9, 2000–2017)
	*Pregnancy length (weeks)*	40 (39.6, 1.7, 26–42)	40 (40.0, 1.6, 32–42)	40 (39.7, 1.7, 26–42)
	***Categorical variables***	***n* (% by birth mode)**	***n* (% by birth mode)**	***n***
**Social Controls**	*Education*			
	Primary/Vocational	34 (83%)	7 (17%)	41
	Secondary	281 (70%)	122 (30%)	403
	University degree	1335 (70%)	584 (30%)	1919
	*Marital status*			
	Cohabitant	378 (70.7%)	157 (29.3%)	535
	Married	1272 (69.6%)	556 (30.4%)	1828
	*Place of residence*			
	City (≥ 100,000 inhabitants)	941 (70.1%)	401 (29.9%)	1342
	Town (< 100,000 inhabitants)	392 (71.8%)	154 (28.2%)	546
	Village	317 (66.7%)	158 (33.3%)	475
**Emotional Factors**	*Planned pregnancy*			
	Unplanned	430 (69.2%)	191 (30.8%)	621
	Planned (trying to get pregnant <12 months)	1054 (70.7%)	436 (29.3%)	1490
	Planned (trying to get pregnant ≥ 12 months)	166 (65.9%)	86 (34.1%)	252
	*Fear of childbirth*			
	Not at all	256 (70.3%)	108 (29.7%)	364
	To a small extent	996 (69.6%)	436 (30.4%)	1432
	Very afraid	398 (70.1%)	169 (29.8%)	567
	*Support during labor*			
	Hospital staff only	111 (48.5%)	118 (51.5%)	229
	Partial personal support—lay companion during part of the labor	240 (39.6%)	366 (60.4%)	606
	Continuous personal support—professional and/or lay companion throughout labor	1299 (85.0%)	229 (15.0%)	1528
**Medical Interventions**	*Oxytocin*			
	No	558 (74.1%)	195 (25.9%)	753
	Yes	1092 (67.8%)	518 (32.2%)	1610
	*Amniotomy*			
	No	1076 (71.4%)	431 (28.6%)	1507
	Yes	574 (67.1%)	282 (32.9%)	856
	*Epidural*			
	No	1278 (77.5%)	372 (22.5%)	1650
	Yes	372 (52.2%)	341 (47.8%)	713

### 2.3 Model comparisons

Our full model included all 12 predictor variables across the three categories (six Controls, three Emotional Factors and three Medical Interventions). We retained the controls in all model comparisons, apart from the intercept-only model. These are variables that might have affected the risk of c-section but which were not the primary focus of our study. Our model comparison approach then focused on adding either or both of the Emotional Factor and Medical Intervention variable categories. Additionally, within each category, we examined which of the predictor variables was most influential and compared models that only included either or both of these variables rather than the category as a whole.

Analyses were conducted in R v. 4.0.4 [36]. Logistic regression models of mode of birth were estimated using the GLM function (family = binomial, link = logit). We then used the MuMIn package [37] to compare alternative models with different subsets of predictors. We used Akaike information criteria (AIC) to compare support for alternative models of mode of childbirth, with lower AIC values indicating greater support for a given model relative to the others [38, 39]. The difference between any two models (Δ) indicates how close they are as competing models, with a Δ ≥ 6 indicating little evidence in the data for the model with the higher value [40].

## 3. RESULTS

Table 2 summarizes the model comparison results. A model with the six sociodemographic control variables (*Age at birth*, *Childbirth year*, *Pregnancy length*, *Education*, *Marital status* and *Place of residence*) was a clear improvement on an intercept-only model, which corresponds to a null model based solely on the percentage of c-sections (Table 2; Model 7 vs. Model 8: AIC Controls = 2858.1, AIC intercept = 2895.9, Δ = 37.8). Among the Control category variables, increasing age at childbirth and increasing pregnancy length were both associated with higher odds of a c-section (Table 3; *Age at birth* (years): OR = 1.05; *Pregnancy length* (weeks): OR = 1.21).

**Table 2.** Model comparison results based on Akaike information criteria (AIC) values. The first model with the lowest AIC value is the best-supported model among those compared. The difference in AIC values between model *i* and the best model is *Δ*_*i*_, number of estimated parameters is *K* and the Akaike weight is *w*_*i*_

Model	AIC	*Δ*_*i*_	*K*	*w*_*i*_
1. Controls^1^ + *Epidural* + *Support during labor*	2253.0	0	12	0.97
2. Controls + Medical Interventions^2^+ Emotional Factors^3^	2260.0	7	18	0.03
3. Controls + *Support during labor*	2383.1	130.1	11	0
4. Controls + Emotional Factors^3^	2387.3	134.3	15	0
5. Controls + *Epidural*	2720.5	467.5	10	0
6. Controls + Medical Interventions	2724.5	471.5	12	0
7. Controls	2858.1	605.1	9	0
8. Intercept-only	2895.9	642.9	1	0

^1^Controls = *Age at birth* + *Childbirth year* + *Pregnancy length* + *Education* + *Marital status* + *Place of residence*

^2^Medical Interventions = *Oxytocin* + *Amniotomy* + *Epidural*

^3^Emotional Factors = *Planned pregnancy* + *Fear of childbirth* + *Support during labor*

**Table 3.** Results from the best model selected. Logistic regression model with mode of birth as response variable (*N* = 2363)

Variable	Coding	Estimate (se)	*Z*-value	OR	95% CI	*P* value
*Age at birth (years)*		0.05 (0.02)	2.70	1.05	1.02–1.08	0.007
*Childbirth year*		0.04 (0.03)	1.50	1.04	1.00–1.09	0.135
*Length of pregnancy (weeks)*		0.19 (0.04)	5.54	1.21	1.15–1.29	<0.001
*Education*	University degree	Reference				
	Primary/vocational	−0.43 (0.48)	−0.89	0.65	0.28–1.39	0.372
	Secondary	−0.09 (0.15)	−0.61	0.91	0.71–1.17	0.541
*Marital status*	Cohabitant	Reference				
	Married	0.03 (0.13)	0.22	1.03	0.83–1.28	0.829
*Place of residence*	City ≥ 100,000 inhabitants	Reference				
	Town < 100,000 inhabitants	−0.14 (0.13)	−1.02	0.87	0.70–1.09	0.307
	Village	0.12 (0.14)	0.87	1.13	0.89–1.43	0.387
*Support during labor*	Hospital staff only	Reference				
	Partial personal support—lay companion during part of the labor	0.13 (0.17)	0.79	1.14	0.87–1.5	0.429
	Continuous personal support—professional and/or lay companion throughout labor	−2.13 (0.17)	−12.84	0.12	0.09–0.16	<0.001
*Epidural*	No	Reference				
	Yes	1.27 (0.11)	11.28	3.55	2.95–4.27	<0.001

Adding Emotional Factors to the Controls led to a vast improvement in the model (Table 2; Model 4 vs. Model 7: AIC Emotional Factors = 2387.3, AIC Controls = 2858.1, Δ = 470.8). Among Emotional Factors, the model that only added *Support during labor* had a lower AIC value than a model with all Emotional Factors (*Support during labor*, *Planned pregnancy* and *Fear of childbirth*) (Table 2; Model 3 vs. Model 4: AIC *Support during labor* = 2383.1, AIC Emotional Factors = 2387.3, Δ = 4.2). Among women with continuous personal support, 85% had vaginal births, compared to women who had partial personal support (39.6%) or support from hospital staff only (48.5%) (Table 1). In the best model (Table 3), continuous personal support during labor was associated with much lower odds of having a c-section than support from hospital staff only (OR = 0.12, CI = 0.09−0.16).

As with Emotional Factors, a model that added Medical Interventions was a large improvement over Controls only (Table 2; Model 6 vs. Model 7: AIC Medical Interventions = 2724.5, AIC Controls = 2858.1, Δ = 133.6). In this case, the inclusion of the *Epidural* variable relative to all Medical Interventions (*Oxytocin*, *Amniotomy* and *Epidural*) was responsible for the improvement in the model (Table 2; AIC *Epidural* = 2720.5, AIC Medical Interventions = 2724.5, Δ = 4.0). Among women with epidurals, 47.8% had a c-section, compared to 22.5% among women without epidurals (Table 1). In the best model (Table 3), women who were given epidurals during labor had increased odds of having a c-section than women who did not (OR = 3.55, CI = 2.95–4.27).

The best models of those compared added both the Emotional Factors and Medical Interventions to the Controls (Table 2; Model 2 vs. Model 7: AIC both sets = 2260.0, AIC Controls = 2858.1, Δ = 598.0). Similar to above, adding only *Support during labor* (from Emotional Factors) and *Epidural* (from Medical Interventions) improved the model even further (Table 2; AIC *Epidural* and *Support during labor* = 2253.0, AIC both sets = 2260.0, Δ = 7.0). Together, those models that incorporate both categories of variables capture all of the weight in the model comparison table (Table 2: Model 1 and Model 2), where the weight expresses the relative likelihood that a given model is the best model among those being compared. Model 1, with just *Epidural* and *Support during labor* added to Controls, is more streamlined than Model 2, which suggests that the other variables in the Emotional Factors and Medical Interventions categories are doing little to improve the model and may just add extra parameters (see discussion in Richards [41]). This is also consistent with an interpretation based on *P* values in a logistic regression table (see Table S1 in Supplementary material).

## 4. DISCUSSION

In this study, we examined potential risk factors for non-elective c-sections among low-risk primiparous women from Poland. Evolutionarily women sought assistance during birth in order to have emotional and physical support. Since humans have evolved to give birth in this context of social support, not having it in modern settings might lead to more complications during birth, and therefore increase rates of c-sections. Evolutionary anthropologists would predict higher rates of c-sections when women lack social support—our results support this, as we have shown that non-elective c-sections were more likely when women lacked continuous personal support during labor. In addition, we have shown that women who were given epidurals were more likely to have c-sections. Taken together, these findings highlight the potential importance of having continuous personal support during labor, as well as including the increased risk of surgical delivery in decision-making about epidural use. Of course, these decisions often interact. Sheila Kitzinger [42] has argued that ‘epidural analgesia may be used instead of emotional support. A midwife who cares for two or three women at the same time is not able to provide good support to each and every of them’. As we have found, continuous personal support, such as through having an individual midwife, may be critical.

### 4.1 Emotional factors during labor

Evolutionary anthropologists would predict that having supportive, familiar and knowledgeable people at the time of labor and delivery is associated with better birth outcomes. In line with this prediction, we have shown that women who received continuous personal support had a lower risk of c-sections. For women in our study, support during labor was given by an individual midwife and/or someone from the woman’s social network. The majority of women in this group had both a midwife and a partner or other family member present throughout labor. The results of our study are consistent with results from other studies [8, 43]. A recent review showed that women who had support not only had lower c-section rates but also were more likely to have spontaneous vaginal birth rather than instrumental vaginal births [8]. In addition, their labors were shorter and they were less likely to report negative childbirth experiences.

In both our evolutionary past and historically, labor was probably most often women-centered, occurring in the presence of female relatives and friends, with men rarely present [1]. In contemporary industrialized settings, medical staff, who women likely do not have strong social ties to, often play a large role in the birth process. This may be considered a mismatch between the social context in which our species has evolved to give birth and the context in which women in industrialized settings currently do give birth [1]. In contemporary Poland, most women choose their partners/husbands as their companions during labor (67.02%) [44]. In contrast, a much smaller number had their mother (2.56%), sister, friend or doula (2.23%) as accompanying person during labor [44]. The remaining 27% of women did not have support other than medical staff in the hospital.

Lower risk of c-section, when women are accompanied by a loved one and/or midwife, may be due to the emotional and social support which they provide. The quality of support provided by the support person may also be important. According to Berkau [45], family births reduce the percentage of medical interventions thanks to the active support of the child’s father. However, studies analysing the experience of childbirth from the father’s perspective indicate that insufficiently prepared and stressed partners may unconsciously influence the work of medical personnel, transfer their emotions to their partners and thus disrupt the course of labor [46]. An important factor in improving perinatal outcomes is therefore the continuous support of the laboring woman by a well-prepared and calm person. In Poland, it is possible to hire a dedicated, professional midwife. Women have time to get to know the midwife during pregnancy. They have had opportunities to discuss the aspects of labor that are most important to the woman such as attitudes toward birth, technology used in hospitals, fears, expectations and the way she would like to give birth. Women will call their midwives when labor starts, and midwives assist the women from the moment they enter the hospital and stay up to 2 h after the birth. This service must be paid out-of-pocket and it costs between EUR 200 and EUR 800, depending on the hospital [47]. This contributes to health inequalities, as only sufficiently wealthy women can afford the support of a professional midwife.

### 4.2 Medical interventions during labor

Medical interventions may also contribute to whether women who intend to give birth vaginally do so. In our study, an epidural is strongly associated with a higher risk of a non-elective c-section, independent of the presence of a support person. This result is consistent with studies from Ireland [48], the Netherlands [49], Australia [50, 51] and Japan [52] that found an increased risk of c-section among women given epidurals, but contradictory to some studies [53–55].

Epidurals are very powerful at relieving labor pain, but they are also associated with longer first and second stages of labor, and a higher risk of oxytocin augmentation, episiotomies, and operative deliveries [48, 53, 55, 56]. Although the mechanisms are not fully understood, longer labor may result from a decrease in uterine activity and slower dilation of the cervix [57], possibly due to a drop in endogenous oxytocin [58]. Moreover, the nerve block may interfere with a woman’s ability to push effectively during contractions [57]. Together, such effects may lead to a determination of failure to progress, and the association between epidurals and c-sections [50], but see also [53].

The type of drug used as epidural analgesia is also important to consider. Intrathecal sufentanil analgesia decreases plasma concentrations of oxytocin and cortisol in women with labor pain during the first stage of labor, but epidural bupivacaine only reduces the cortisol concentration [59]. Unfortunately, our study lacks information on the specific drugs women received during labor, and women gave birth in many different hospitals around Poland, each with its own protocol for analgesia during labor.

We found that women who were given oxytocin did not have an elevated risk of c-section. Although there may be exceptions [51], our study is generally consistent with the others (including a meta-analysis) that do not find an increased risk of c-sections with oxytocin use either for induction or augmentation of labor [60–62]. It is known that individual interventions (e.g. labor induction, epidural anesthesia) and a cascade of interventions throughout labor may disrupt hormonal physiology and introduce risks to the woman or her baby, both in the short and long term [58]. The use of epidural has been associated with more frequent use of oxytocin to speed up labor [52, 63, 64]. Epidural during labor may interfere with the release of oxytocin, which may prolong labor and, as a result, increase the need to stimulate labor with artificial oxytocin, the action of which is slightly different from that of natural oxytocin [10, 65].

### 4.3 Policy implications

Modern medical interventions during labor have significantly improved maternal and neonatal survival rates at the population level, but these procedures also include serious risks, and so their overuse is also not in the best interest of individuals or public health [14, 66]. Since 2015, all Polish women have been able to request free-of-charge epidural during labor. There was hope that labor without pain would result in lower c-section rates. However, we do not observe this in national statistics—rates continued to rise after this policy was enacted [28]. Nevertheless, the demand for epidurals is high. Based on information collected from over 8000 women who gave birth in 2017 and 2018, 23.7% of women in Poland received an epidural [67] and more may have wanted it—13% of women declared that they were not given an epidural even though they requested it.

We are not arguing here that women should be limited in their choices for pain management, but rather that disrupting this sensory system may have unintended consequences about which women should be informed. It is especially important to help women avoid unnecessary c-sections in first pregnancies as this elevates the risk of c-sections in subsequent pregnancies [21]. Our findings suggest that lack of personal emotional support as well as medical interventions that may disrupt the hormonal physiology during birth, may contribute to rapidly rising rates of cesarean. Good national-level data are needed to identify whether rising c-sections rates reflect changes in decision-making about the medical need for the procedure, in contrast to potentially avoidable procedures due to a mismatch between women’s needs during labor and current medical practice.

One approach that is used to systematically examine c-sections across different types of births is called Robson’s Ten Group Classification System, which classifies all deliveries into ten mutually exclusive groups based on a set of predefined obstetric parameters (e.g. based on parity and fetal position) [68]. This allows for the identification of the groups that make significant contributions to the overall c-section rate, as well as those groups with substantial heterogeneity in rates across regions [69]. This system is recommended by the WHO and widely used in many other countries, but it is not used in Poland as a standard procedure for assessing and monitoring c-section rates in hospitals. Its use could help to identify groups in which c-sections are used most widely on a national level and then to prepare targeted educational interventions that would contribute to steps that could reduce rising c-section rates in Poland. Our present study’s results apply to groups including nulliparous singleton births with cephalic presentations, although we would predict that multiparous women will also benefit from continuous social support.

## 5. CONCLUSIONS

In Poland, c-section rates rose rapidly in the last decade from below 20% in 2007 to 39.3% in 2017 [28]. This is one of the highest c-section rates among OECD countries, despite many improvements over this time to Polish hospitals and very good standards of perinatal care [67]. Our study is among the first population-based studies of the mode of birth in Poland and shows that lack of continuous personal support and the use of epidurals both increase the odds of a c-section over vaginal birth. Solitary birth is rare among humans, and the childbirth process appears to have long-included the presence of others who assist laboring women [1]. Unfortunately, sufficient and continuous social and emotional support is often lacking in modern medical settings, which may contribute to complications during labor including obstetrical difficulties such as c-sections that might otherwise be avoided. Results such as ours are key to better understanding avenues for reducing c-sections among low-risk women who are giving birth for the first time, for example by prioritizing continuous social and emotional support during labor and delivery.

## Supplementary Material

eoad013_suppl_Supplementary_Material

## Data Availability

The datasets analysed during the current study are available from the corresponding author upon reasonable request.
