# Supplementary material for: Emotional factors, medical interventions and mode of birth among low-risk primiparous women in Poland
Source: Evol Med Public Health. 2023 May 14;11(1):139–48. doi: 10.1093/emph/eoad013 (PMC10224696; doi:10.1093/emph/eoad013)
Supplement: eoad013_suppl_Supplementary_Material [file eoad013_suppl_supplementary_material.docx]

Supplementary materials

**Emotional factors, medical interventions and mode of birth among low-risk primiparous women in Poland**

Table S1. Results from logistic regression model with mode of birth as response variable (N = 2363). Full model.

| **Variable** | **Coding** | **Estimate (se)** | **Z-value** | | **P-value** | |
| --- | --- | --- | --- | --- | --- | --- |
| *Age at birth (years)* |  | 0.04 (0.02) | 2.54 | | 0.01 | |
| *Childbirth year* |  | 0.04 (0.03) | 1.56 | | 0.12 | |
| *Pregnancy length (weeks)* |  | 0.19 (0.04) | 5.26 | | <0.001 | |
| *Education* | University degree | Reference |  | |  | |
|  | Primary/vocational | -0.45 (0.48) | -0.92 | | 0.36 | |
|  | Secondary | -0.12 (0.16) | -0.75 | | 0.46 | |
| *Marital status* | Cohabitant | Reference |  | |  | |
|  | Married | 0.05 (0.14) | 0.38 | | 0.71 | |
| *Place of residence* | City ≥ 100,000 inhabitants | Reference |  | |  | |
|  | Town < 100,000 inhabitants | -0.15 (0.14) | -1.07 | | 0.28 | |
|  | Village | 0.11 (0.14) | 0.77 | | 0.45 | |
| *Planned pregnancy* | Unplanned | Reference |  | |  | |
|  | Planned, trying to get pregnant <12 months | -0.12 (0.13) | -0.90 | | 0.37 | |
|  | Planned, trying to get pregnant ≥ 12 months | 1.15 (0.20) | 0.76 | | 0.45 | |
| *Fear of childbirth* | Not at all | Reference |  | |  | |
|  | To a small extent | -0.07 (0.15) | -0.44 | | 0.66 | |
|  | Very afraid | -0.17 (0.17) | -0.99 | | 0.32 | |
| *Support during labor* | Hospital staff only | Reference |  | |  | |
|  | Partial personal support – lay companion during part of the labor | 0.11 (0.17) | 0.64 | 0.52 | |  |
|  | Continuous personal support – professional and/or lay companion throughout labor | -2.16 (0.17) | -12.89 | <0.001 | |  |
| *Oxytocin* | No | Reference |  | |  | |
|  | Yes | 0.11 (0.12) | 0.91 | | 0.37 | |
| *Amniotomy* | No | Reference |  | |  | |
|  | Yes | 0.05 (0.11) | 0.46 | | 0.65 | |
| *Epidural* | No | Reference |  | |  | |
|  | Yes | 1.24 (0.12) | 10.86 | | <0.001 | |
